# Classification of Plant Leaf Diseases Based on Improved Convolutional Neural Network

**DOI:** 10.3390/s19194161

**Published:** 2019-09-25

**Authors:** Jie Hang, Dexiang Zhang, Peng Chen, Jun Zhang, Bing Wang

**Affiliations:** 1 School of Electrical Engineering and Automation, Anhui University, Hefei 230601, China; ahu0086@163.com (J.H.); 13096@ahu.edu.cn (D.Z.); wwwzhangjun@163.com (J.Z.); 2 Institutes of Physical Science and Information Technology, Anhui University, Hefei 230601, China; 3 School of Electrical and Information Engineering, Anhui University of Technology, Ma’anshan 243032, China

**Keywords:** plant leaf disease, convolutional neural network, inception structure, squeeze-and-excitation module, global average pooling

## Abstract

Plant leaf diseases are closely related to people’s daily life. Due to the wide variety of diseases, it is not only time-consuming and labor-intensive to identify and classify diseases by artificial eyes, but also easy to be misidentified with having a high error rate. Therefore, we proposed a deep learning-based method to identify and classify plant leaf diseases. The proposed method can take the advantages of the neural network to extract the characteristics of diseased parts, and thus to classify target disease areas. To address the issues of long training convergence time and too-large model parameters, the traditional convolutional neural network was improved by combining a structure of inception module, a squeeze-and-excitation (SE) module and a global pooling layer to identify diseases. Through the Inception structure, the feature data of the convolutional layer were fused in multi-scales to improve the accuracy on the leaf disease dataset. Finally, the global average pooling layer was used instead of the fully connected layer to reduce the number of model parameters. Compared with some traditional convolutional neural networks, our model yielded better performance and achieved an accuracy of 91.7% on the test data set. At the same time, the number of model parameters and training time have also been greatly reduced. The experimental classification on plant leaf diseases indicated that our method is feasible and effective.

## 1. Introduction

With the rapid development of computer technology, traditional machine learning methods have been applied in plant diseases prediction more and more widely. With the popularity of machine learning algorithms in computer vision, in order to improve the accuracy and speed of diagnostic results, researchers have studied automated plant disease diagnosis based on traditional machine learning algorithms, such as random forest, k-nearest neighbor and support vector machine (SVM) [1,2,3]. Tan et al. established a multi-layer BP neural network model to realize the disease identification of soybean leaves, by calculating the chromaticity values of the leaves [4]. By extracting the color and texture characteristics of grape disease leaves, Tian et al. used a support vector machine (SVM) recognition method which achieved better results than the neural network [5]. Wang et al. developed a discriminant analysis method to identify cucumber lesions, by extracting the color, shape and texture features of leaf lesions, as well as combining with environmental information [6]. Zhang et al. also extracted the color, shape and texture features of lesion after lesion segmentation, and then used them to identify five types of corn leaves by K-nearest neighbor (KNN) classifier [7].

Spraying pesticides is a key step in plant growing. Ron et al. developed a robotic sprayer platform with the collaboration of remote operators for detecting targets and completing spraying tasks. They reported that it can prevent 30% to 35% of crop losses [8]. Autonomous selectivity for spraying pesticides can be used to reduce production costs in agriculture, using an auto-driving robot to detect plants in the crop area and to perform selective injection [9]. Moreover, plant disease detection can be based on spectroscopy and imaging processes. Xie et al. proposed an automatic classifier with multi-level learning features for field crop pests [10]. Sindhuja developed a ground-based sensor system to help monitor the status of plant health and diseases in field conditions [11].

Li et al. proposed an improved deep learning pipeline for automatic localization and count of agricultural crop pests, which integrates a convolutional neural network (CNN) of ZF (Zeiler and Fergus model) and a region proposal network (RPN) with Non-Maximum Suppression (NMS) [12]. Yang et al. proposed a new identification method for rice diseases based on deep convolutional neural networks [13]. The model was trained to identify 10 common rice diseases and tested on a dataset containing 500 natural images of diseased and healthy rice leaves and stems captured from rice fields. Under the 10-fold cross-validation strategy, the CNN-based model achieved an accuracy of 95.48%. Xia et al. proposed a convolutional neural network model to solve the problem of multi-classification of crop insects and achieved a heightened accuracy [14]. Sun et al. proposed an improved convolutional neural network which achieved an accuracy of 99.35% on a test dataset containing 54,306 images, which is composed of 26 diseases in 14 different plants [15]. Moreover, the Pascal VOC (Visual Object Classes) Challenge [16] and the recent ImageNet Large Scale Visual Recognition Challenge (ILSVRC) [17] based on the ImageNet dataset [18], have been widely used as benchmarks for many visualization issues in computer vision, including object classification. In 2012, a convolutional neural network, called AlexNet (Alex’s Network) [19], reduced the top5 error rate of the 1000 categories of classified images to 16.4% on ImageNet. 

This work focused on five different convolutional neural network architectures, AlexNet, VGGNet (Visual Geometry Group’s Network) [20], GoogLeNet (Google’s Network) [21], ResNet (Residual Network) [22] and SENet (Squeeze-and-Excitation Network) [23], which participated in ILSVRC from 2012 to 2017, and achieved good classification results. There are still some challenges in plant leaf disease classification, which are as follows:

(1) Limited by experimental conditions, such as current platform and hardware, a large CNN network will cost a long training time and have a slow convergence rate;
(2) Long training convergence time will cause the final classification accuracy to decrease.

To shorten long training convergence time, decrease enormous parameters of most current network models, and increase recognizing accuracy, this paper proposes an integrated method. It adopts the inception structure to fuse the extracted high-level features, the Squeeze-and-Excitation module to perform feature re-calibration for weighting the features in the channel of CNN, and global average pooling instead of the fully connected layer. The experimental results show that our method is effective in the classification and identification of plant leaf diseases. Compared with other traditional convolutional neural networks, our model achieved the highest classification accuracy rate of 91.7% on our plant leaf disease dataset.

## 2. Materials and Methods

### 2.1. Data Preprocessing and Augmentation

**Figure 1:**
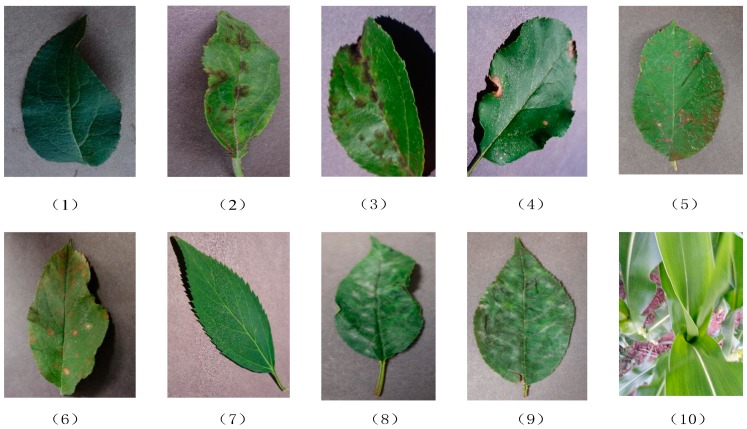
Sample images of 10 leaf diseases. (**1**) Apple healthy (AH); (**2**) Apple Scab general (ASG); (**3**) Apple Scab serious (ASS); (**4**) Apple Frogeye Spot (AFS); (**5**) Cedar Apple Rust genera (CARG)l; (**6**) Cedar Apple Rust serious (CARS); (**7**) Cherry healthy (CH); (**8**) Cherry Powdery Mildew general (CPMG); (**9**) Cherry Powdery Mildew serious (CPMS); (**10**) Corn healthy (CH).

We collected 10 kinds of disease leaf images from a library of plant leaf diseases (https://challenger.ai/), where digital color cameras were used for capturing diseased blade images with a resolution width of 256 and an unfixed length, as shown in Figure 1. Because some types of leaf diseases are confusing and unidentifiable, only 10 types of blade data were selected for our research. As for the images of plant corn, we tried to adopt the image class to verify the identification and showed the generalization ability of different CNN network structures for different types of leaf diseases. The diseased parts of apple and cherry leaves are similar, and the degrees of leaf diseases in different disease levels are also similar. It is more practical for our research. Compared with other types of leaf diseases, these diseased leaves can better reflect the distinguishing ability of disease areas for different CNN structures, and can better compare the ability of different CNN structures in leaf classification.

First, all leaf disease images were adjusted so that the length and width of the image were the same, which were resized to 224 × 224. Resizing images to 224 × 224 before inputting images into different networks is done to adapt different pre-training CNN structures. Then, because some leaf disease types contains less images than others and the collection of leaf disease images are random, images of these disease types were horizontally and vertically flipped. The leaf diseases are Cedar Apple Rust—serious, Cherry Powdery Mildew—general, and Cherry Powdery Mildew—serious. Thus, the leaf disease data set was expanded to prevent redundancy of the data set, ensure the validity of image data, and make the classifier balanced. After the data augmentation, the plant leaf disease dataset contained 6108 images, of which 5588 were for the training set and 520 were for the test set. Table 1 lists the number of images for each disease class.

### 2.2. Convolutional Neural Network (CNN)-Based Method

#### 2.2.1. CNN Overall Architecture

**Figure 2:**
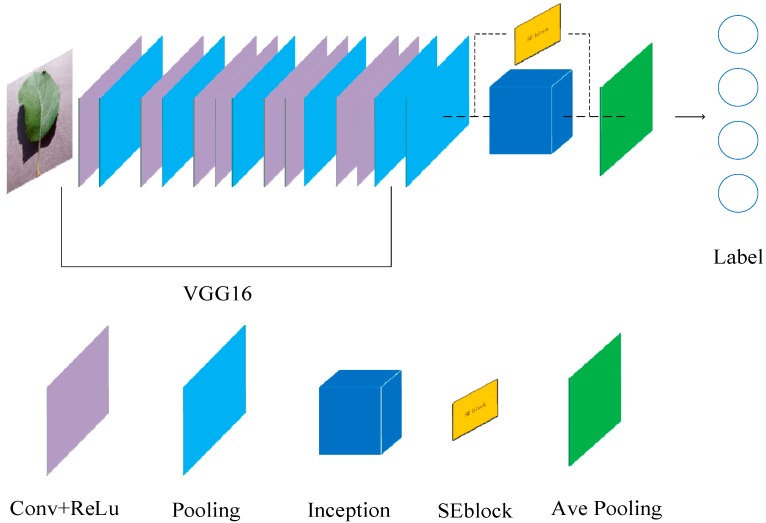
The structure of the proposed convolutional neural network (CNN).

Our deep learning-based network consists of VGG16 convolutional layers as well as the combination of Squeeze-and-Excitation (SE) module and Inception structure. The first five convolutional layers are based on the VGG16 model for self-learning low-to-high features of training images, where deeper convolutional layers reduce more resolution of feature maps, and extract more abstract high-level features. Then, the max pooling layer is used to filter the noise of the feature maps generated by the previous convolutional layer. Inception structure performs feature fusion, broadens the ability of acquiring features on feature maps, and extracts the best distinguishing features based on multi-dimensional analysis. The embedded SE module, re-calibrating the original features in the channel dimension, is used to replace the fully connected layer with the largest average pooling layer, reduce the training parameters as well as quickening the convergence of the model, and thus improving the classification accuracy of the model. The network structure of the improved model and related parameters are shown in Figure 2 and Table 2, respectively.

The five convolutional layers are based on VGG16 pre-training model, which determines which layers of the original network have to be frozen during the pre-training phase, and which layers are allowed to continue learning at a certain learning rate. Usually, the first several layers are frozen because the low-level features can better adapt to various problems. This work used a stochastic gradient descent optimization method to train the model on our own data set. The initial learning rate was set to 0.001, while momentum and weight attenuation were set to 0.0005 and 0.9, respectively. The Dropout layer [24] was used in our experiments to prevent over-fitting in training and make the model more effective.

#### 2.2.2. GoogLeNet’s Inception 

**Figure 3:**
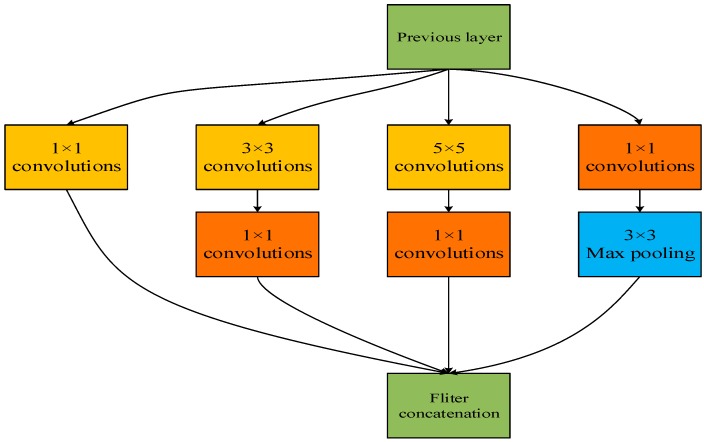
Inception structure model.

Inception module is the main component of GoogLeNet network. The Inception structure embeds multi-scale information and gathers features from different receptive fields to improve identification performance. It maintains the sparse structure, increases the depth and broadens the width of the network, therefore it reduces not only over-fitting but also free parameters. Figure 3 shows that the Inception module uses three different convolution kernels, 1 × 1 convolution, 3 × 3 convolution, 5 × 5 convolution as well as a 3 × 3 max pooling layer. It extracts three different scale features to increase the diversity of features, involving both macroscopic features and microscopic features. The purpose of the pooling layer is to preserve the primitive input information. The module splices the extracted features in the channel dimension and outputs a multi-scale feature map by concatenating these convolutional and pooling layers together. 

#### 2.2.3. Global Average Pooling (GAP)

**Figure 4:**
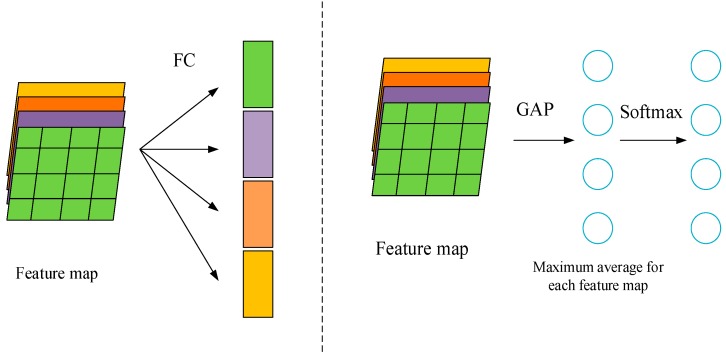
Comparison of the fully connected layer and the global averaged pooled layer.

The fully connected network has always been the standard configuration of the CNN network. However, too many parameters in the fully connected layer slows down the training speed of the network and makes it easy to be overfitting. The idea of global average pooling (GAP) [25] is to globally average the entire pixels of each feature map, and get an output for each feature map. The vector that is composed of these output features will be directly sent to softmax for classification. Figure 4 shows the comparison of the fully connected layer and the global averaged pooled layer.

#### 2.2.4. Squeeze-and-Excitation Module

**Figure 5:**
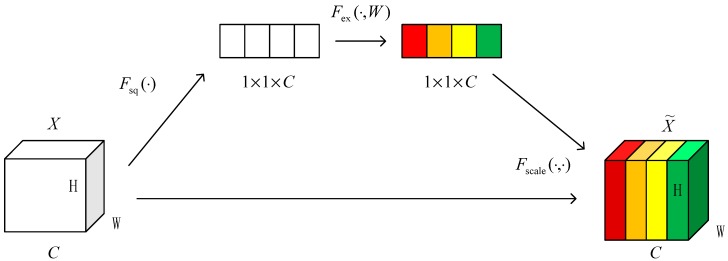
Squeeze-and-Excitation (SE) module.

Figure 5 is a schematic diagram of the SE module, which omits the previous series of convolutions in the original SE module. Given an input *X*, the number of feature channels is *C*. Unlike the traditional CNN, three operations are taken to recalibrate previously obtained features.

The first one is Squeeze operation. Suppose that the inputs are *X* = (*X*_1_, *X*_2_, …, *X_C_*), *X_C_* ∈ *R^H×W^*. Formally, a statistic *z* ∈ *R^C^* is generated by shrinking *X* through its spatial dimensions *H* × *W*. The *c*-th element of *Z* is calculated by:

(1) $$Z_{c}=F_{sq}(X_{c})=\frac{1}{W\times H}\sum_{i=1}^{W}\sum_{j=1}^{H}X_{c}(i,j).$$

Therefore, the Squeeze operation converts the input of H×W×C into an output of 1×1×C, corresponding to the *F_sq_* operation in Figure 5. The result of this step is equivalent to the numerical distribution of the *C* feature maps of the layer, or global information. The output *Z_c_* can be thought of as the description of a set of local descriptors for the entire channel map. 

The second operation is the Excitation operation. It can represent the convolution and activation operations, which employ a simple gating mechanism with a sigmoid activation:

(2) $$S=F_{ex}(Z,W)=\sigma (g(Z,W))=\sigma (W_{2}\delta (W_{1}*Z)),$$

where, *δ* refers to the ReLu function, and the output *Z* can be thought of as a set of local descriptors for the entire channel map, $W_{1}\in R^{\frac{C}{r}\times C}$ and $W_{2}\in R^{C\times \frac{C}{r}}$. In order to control the complexity and generalization of the model, the embedding mechanism of the model is parameterized by two nonlinear fully connected layers.

Finally, a reweight operation regards the weight of the output of Excitation as the importance of each feature channel after feature selection, and then weights previous features by channel weighting to complete the pair in the channel dimension. The output of the block is obtained by rescaling *X* with the activations *s*:

(3) $${\tilde{x}}_{C}=F_{scale}(x_{C},s_{C})=s_{C}\cdot x_{C},$$

where, $F_{scale}(x_{c},s_{c})$ refers to channel-wise multiplication between the scalar *s_C_* and the feature map *x_C_* ∈ *R^H×W^*, and $\tilde{x}=|{\tilde{x}}_{1},{\tilde{x}}_{2},\ldots {\tilde{x}}_{C}|$.

**Figure 6:**
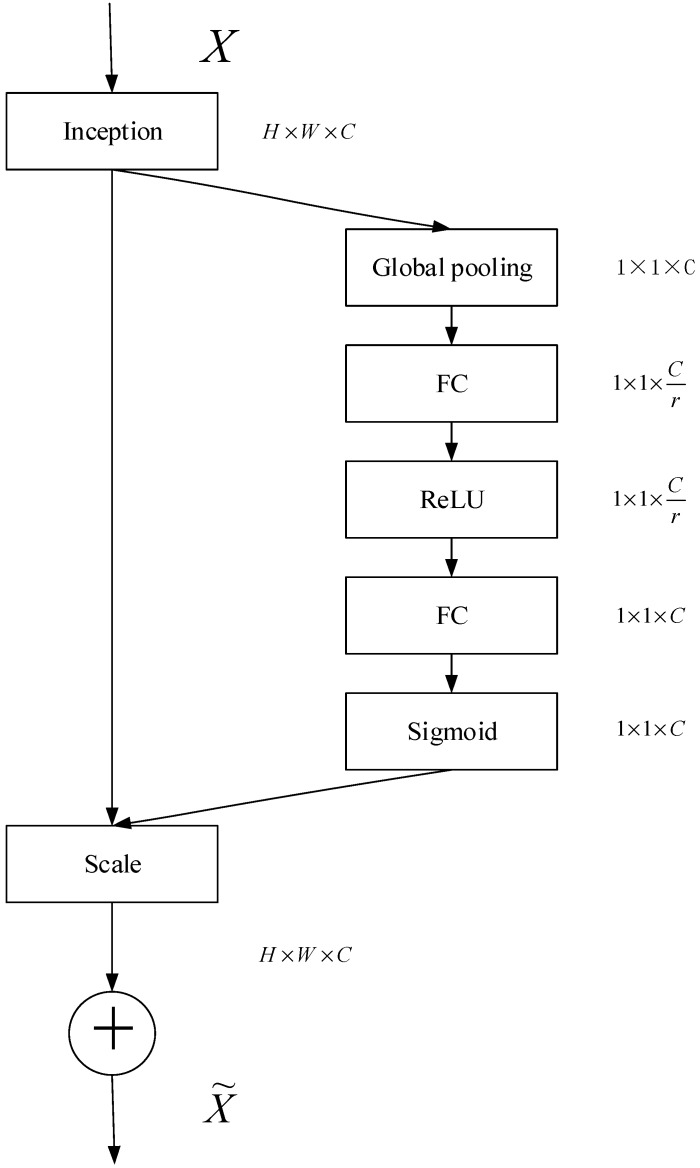
The combined structure of the SE module and the Inception structure.

The SE module can be embedded in the Inception and standard network architecture of ResNet, as shown in Figure 6. Figure 6 is a combination structure of the SE module and the Inception module.

## 3. Experiments and Results 

The experiments were performed on an Ubuntu workstation with CPU i7-8700k and RAM 32G, accelerated by two NVIDIA GTX 1080TI GPUs. All of our experiments were implemented by Caffe, a deep learning open source framework [26]. Moreover, accuracy rate was used to evaluate the performance of network models. The accuracy rate refers to the proportion of the number of corrected positive predictions to that of the whole positive predictions. It can be expressed as:

(4) $$Accuracy=\frac{N_{TP}}{N_{TP}+N_{FP}},$$

where, *N_TP_* is the number of corrected positive predictions, and *N_FP_* is the number of wrongly positive predictions.

### 3.1. Effects of the Feature Extraction Network

The most important metric we considered is the average accuracy of the test set. Table 3 lists the experimental accuracy, model size and training time for several commonly used deep learning CNN architectures, as well as the results of our method.

**Figure 7:**
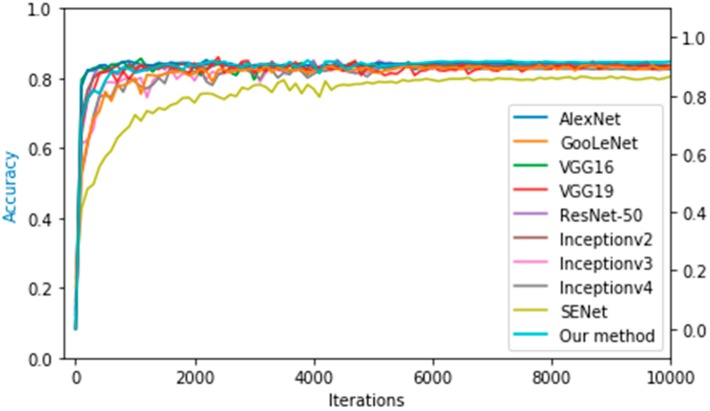
Trends in the accuracy of different CNN models on the test set.

The first observation from Table 3 is that different convolution depths make the trained model produce different classification results on the test set. In general, more convolutional layers can learn more complex features from original images. Shallow CNNs such as AlexNet achieved an accuracy of 0.894 on the test set, while the deep networks VGG16, VGG19, ResNet-50, and Inceptionv2 yielded accuracies of 0.905, 0.903, 0.901 and 0.903 on the test set, respectively. Compared to other networks, our network is relatively shallow, but achieves higher accuracy on the test set. One possible reason is in that shallow network has a relatively good generalization compared to deep ones. The other reason is because of the use of the Inception module to broaden the network and combine the multi-scale feature information, as well as the use of the SE module to merge the feature channel into the Inception module and thus weighted and recalibrated features. As a result, our network achieved a maximum accuracy of 91.7% on the test set. Figure 7 shows the trends of accuracy of different CNN models on the test set.

### 3.2. Comparison of Model Size for Different Network Models

From the comparison of model size of different models in Table 2, we can get an intuitive observation that the larger the size of the CNN model, the more parameters the CNN had, and the longer the training time. The size of the AlexNet, VGG16, and VGG19 training models were 217 MB, 537.2 MB, and 558.4 MB, respectively. The large model size is because the last three layers of these network structures are all fully connected, which causes the number of the trained network model size to be larger than that of other deep learning models. On the contrary, GoogLeNet, Inceptionv2, and Inceptionv3 with Inception structure greatly reduce the size amount to 47.1 MB, 45.1 MB, and 87.3 MB, respectively. The size of our model is 57.3 MB, which is greatly reduced compared with VGG16 and VGG19. The reason is that our model used the Inception structure and the global average pooling instead of the last three-layer fully connected layer. This structure can avoid the requirement of a large number of weight parameters, reduce the size of the CNN model and solve the problem of large memory occupancy and slow convergence in the training CNN model.

### 3.3. Comparison of Training Time for Different Network Models

The general CNN model linearly converts all extracted feature maps into 4096-dimensional feature vectors after convolutional and pooled layers, and classifies leaf diseases by softmax layer. Table 4 shows the training time of the forward propagation and backpropagation processes for different CNN models and improved models. As can be seen from Table 4, our model performs a forward propagation rate of 0.038 s, which means that the time required to test a picture is 0.038 s. Compared with other CNN models, our model has a faster advantage in the forward propagation time.

### 3.4. Loss Function and Confusion Matrix of Our Network

**Figure 8:**
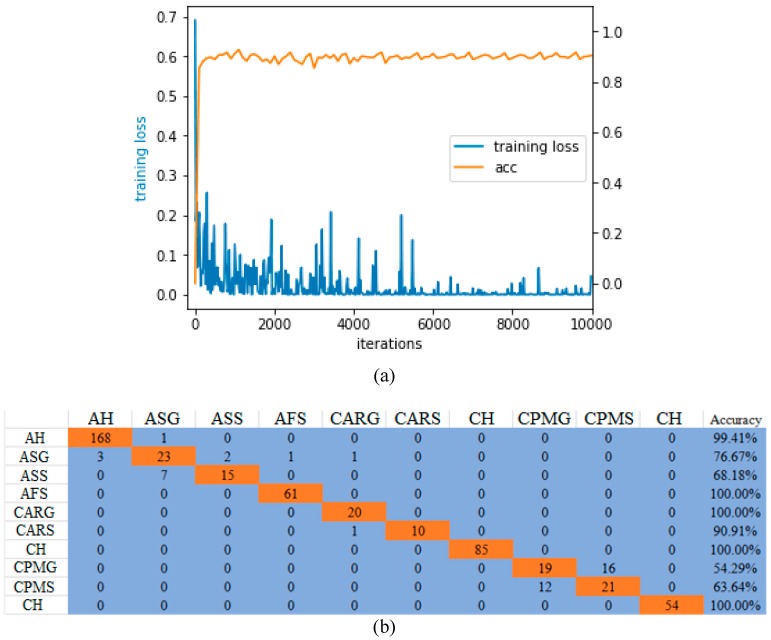
Trend graph of the loss function (**a**) and confusion matrix (**b**). (**1**) Apple healthy (AH); (**2**) Apple Scab general (ASG); (**3**) Apple Scab serious (ASS); (**4**) Apple Frogeye Spot (AFS); (**5**) Cedar Apple Rust genera (CARG)l; (**6**) Cedar Apple Rust serious (CARS); (**7**) Cherry healthy (CH); (**8**) Cherry Powdery Mildew general (CPMG); (**9**) Cherry Powdery Mildew serious (CPMS); (**10**) Corn healthy (CH).

**Figure 9:**
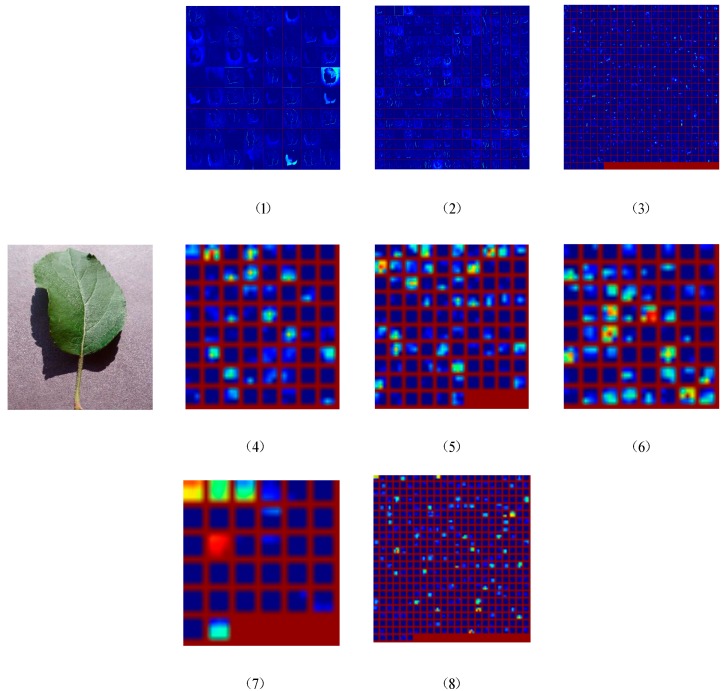
Visualization of feature map from each layer for a sample leaf. (**1**) conv1_1 (see Table 2), (**2**) conv3_1, (**3**) conv5_1, (**4**) inception_ 1 × 1, (**5**) inception_ 3 × 3, (**6**) inception_ 5 × 5, (**7**) inception_ pool, (**8**) pool7.

From Figure 8a, it can be concluded that our model tends to converge (blue curve), and the final accuracy rate is stable at 91.7% (orange curve), achieving a better classification result. Accuracy is an unreliable performance metric for evaluating the classification model because it can produce misleading results when the sample numbers of different classes in the data set are unevenly distributed. Moreover, the average accuracy of all categories is an accurate indicator for the model on the test set. In other words, the categories that are difficult to classify will be improved by the easily classified categories. The confusion matrix is the degree to which a classification model is accurate for each classification category. From the confusion matrix in Figure 9b, we can conclude that for some difficult-to-classify plant leaf diseases, the classification accuracy of such single-category on the test set is low, because the diseased region in each leaf is too small and the number of different grades of leaf disease is different. Therefore, it is difficult to be classified and identified by model. For instance, leaves of “Cherry Powdery Mildew – general” and those of “Cherry Powdery Mildew – serious” are difficult to be classified, because most of the regions in these leaves are very similar. The confusion matrix of the last experiment showed that the accuracy of disease recognition for corn is 100%, which did not interfere with other types of leaf diseases in classification.

### 3.5. Visualization of Feature Extraction

Figure 9 visualizes a list of feature extractions after different layers of our network. The visualization of the network model can help us to intuitively understand the classification model. The ideal feature map of CNN should be sparse and contain typical local information. Through the visualization of the model, we can understand what features each layer of CNN learns, which can be used to adjust network parameters to improve the accuracy of the model. As a result, it provides a better understanding of how the CNN network learns the characteristics of the input image by visualizing various convolutional layers. We found that the features learned by CNN are hierarchical. The higher the level is, the more the specific features are presented. Moreover, the higher the dimensional feature maps correctly classify the images, the greater the effect presents. Specifically, the deep layer (Figure 9(7) or (8)) presents some edge corners and abstract features of colors, and the shallow feature map (Figure 9(1) or (2)) responds to the color information of the corners and other edges. The feature map of the middle layer (Figure 9(3), (4), (5), or (6)) has more complex invariance, captures similar textures, and has more layers for feature extraction. The high-level feature map shows the salient pose of the entire image after the extraction of the high-level abstract features.

## 4. Conclusions

This paper proposed an improved structure of convolutional neural networks for the identification and classification of a large dataset of different plant leaf diseases. Based on the traditional five-layer convolutional model of VGG16, the final, fully connected layer of VGG16 was replaced with Inception and SE modules, which can improve the classification accuracy of the model on the plant leaf disease dataset. Moreover, the global pooling layer can shorten the training time and parameter memory requirements, and also improve the generalization ability of the model. As a result, our method achieved the highest classification accuracy of 91.7% on the test set of plant leaf diseases. Compared with some other CNN methods, it has better adaptability to the change of image spatial position, showing better robustness to identify different diseases of various plant leaves, not limited to different diseases of the same plant.

## Figures and Tables

**Table 1:** Statistics of the plant leaf disease dataset.

Class	Number of Training Images (Before Data Augmentation)	Number of Training Images (After Data Augmentation)	Number of Testing Images
Apple healthy	1185	1185	169
Apple Scab general	844	844	30
Apple Scab serious	596	596	22
Apple Frogeye Spot	427	427	61
Cedar Apple Rust general	142	142	20
Cedar Apple Rust serious	40	160	11
Cherry healthy	598	598	85
Cherry Powdery Mildew general	162	648	35
Cherry Powdery Mildew serious	153	612	33
Corn healthy	376	376	54
Total	4523	5588	520

**Table 2:** Related parameters of the convolutional neural network (CNN)-based model.

Type	Size/Stride	Output Size
Conv1 (Convolutional layer 1)	3 × 3/1	64 × 224 × 224
Pool1/max	3 × 3/1	64 × 112 × 112
Conv2	3 × 3/1	128 × 112 × 112
Pool2/max	3 × 3/1	128 × 56 × 56
Conv3	3 × 3/1	256 × 56 × 56
Pool3/max	3 × 3/1	256 × 28 × 28
Conv4	3 × 3/1	512 × 28 × 28
Pool4/max	3 × 3/1	512 × 14 × 14
Conv5	3 × 3/1	512 × 14 × 14
Pool5/max	3 × 3/1	512 × 7 × 7
Pool6/max	3 × 3/1	512 × 3 × 3
Inception	-	256 × 3 × 3
Pool7/ave	3 × 3/1	256 × 1 × 1
Dropout	-	256 × 1 × 1
Linear	-	10 × 1 × 1
Softmax	-	10

**Table 3:** Classification accuracy (in percent) comparison of CNN-Based models.

CNN	Accuracy	Model Size	Training Time
AlexNet	0.894	217 MB	1140.15 s
GoogLeNet	0.898	47.1 MB	332.228 s
VGG16	0.905	537.2 MB	1960.2 s
VGG19	0.903	558.4 MB	5411.31 s
ResNet-50	0.901	94.3 MB	2101.19 s
Inceptionv2	0.903	45.1 MB	2187.3 s
Inceptionv3	0.901	87.4 MB	6438.72 s
Inceptionv4	0.89	165 MB	5787.9 s
SENet	0.875	220.8 MB	1794.78 s
**Our method**	**0.917**	**57.3 MB**	**961.1 s**

**Table 4:** Comparison of training time for different CNN models.

CNN	Forward Pass	Backward Pass	Total Time
AlexNet	0.052 s	0.061 s	1140.15 s
GoogLeNet	0.013 s	0.019 s	332.228 s
VGG16	0.047 s	0.014 s	1960.2 s
VGG19	0.167 s	0.371 s	5411.31 s
ResNet-50	0.096 s	0.114 s	2101.19 s
Inceptionv2	0.102 s	0.117 s	2187.3 s
Inceptionv3	0.301 s	0.342 s	6438.72 s
Inceptionv4	0.258 s	0.321 s	5787.9 s
SENet	0.116 s	0.179 s	1794.78 s
Our method	0.038 s	0.053 s	961.1 s
